# Exploring common pathogenic association between Epstein Barr virus infection and long-COVID by integrating RNA-Seq and molecular dynamics simulations

**DOI:** 10.3389/fimmu.2024.1435170

**Published:** 2024-09-26

**Authors:** Ayesha Kanwal, Zhiyong Zhang

**Affiliations:** ^1^ MOE Key Laboratory for Cellular Dynamics and Division of Life Sciences and Medicine, University of Science and Technology of China, Hefei, Anhui, China; ^2^ Department of Physics, University of Science and Technology of China, Hefei, Anhui, China

**Keywords:** RNA-Seq, long-COVID, hub-genes, EBV-reactivation, bioflavonoids, molecular docking, molecular dynamics simulation

## Abstract

The term "Long-COVID" (LC) is characterized by the aftereffects of COVID-19 infection. Various studies have suggested that Epstein–Barr virus (EBV) reactivation is among the significant reported causes of LC. However, there is a lack of in-depth research that could largely explore the pathogenic mechanism and pinpoint the key genes in the EBV and LC context. This study mainly aimed to predict the potential disease-associated common genes between EBV reactivation and LC condition using next-generation sequencing (NGS) data and reported naturally occurring biomolecules as inhibitors. We applied the bulk RNA-Seq from LC and EBV-infected peripheral blood mononuclear cells (PBMCs), identified the differentially expressed genes (DEGs) and the Protein–Protein interaction (PPI) network using the STRING database, identified hub genes using the cytoscape plugins CytoHubba and MCODE, and performed enrichment analysis using ClueGO. The interaction analysis of a hub gene was performed against naturally occurring bioflavonoid molecules using molecular docking and the molecular dynamics (MD) simulation method. Out of 357 common genes, 22 genes (CCL2, CCL20, CDCA2, CEP55, CHI3L1, CKAP2L, DEPDC1, DIAPH3, DLGAP5, E2F8, FGF1, NEK2, PBK, TOP2A, CCL3, CXCL8, DEPDC1, IL6, RETN, MMP2, LCN2, and OLR1) were classified as hub genes, and the remaining ones were classified as neighboring genes. Enrichment analysis showed the role of hub genes in various pathways such as immune-signaling pathways, including JAK-STAT signaling, interleukin signaling, protein kinase signaling, and toll-like receptor pathways associated with the symptoms reported in the LC condition. ZNF and MYBL TF-family were predicted as abundant TFs controlling hub genes' transcriptional machinery. Furthermore, OLR1 (PDB: 7XMP) showed stable interactions with the five shortlisted refined naturally occurring bioflavonoids, i.e., apigenin, amentoflavone, ilexgenin A, myricetin, and orientin compounds. The total binding energy pattern was observed, with amentoflavone being the top docked molecule (with a binding affinity of –8.3 kcal/mol) with the lowest total binding energy of −18.48 kcal/mol. In conclusion, our research has predicted the hub genes, their molecular pathways, and the potential inhibitors between EBV and LC potential pathogenic association. The *in vivo* or *in vitro* experimental methods could be utilized to functionally validate our findings, which would be helpful to cure LC or to prevent EBV reactivation.

## 1 Introduction

After the declaration of the coronavirus disease 2019 (COVID-19) pandemic, the world continues to face its aftereffects (1). There is considerable knowledge available on COVID-19 that focuses on the acute illnesses linked to it (2–4); overall, it is also evident that there are tremendous long-term consequences (5, 6) associated with it. Even after recovering from COVID-19, there has been a population of patients who are continuously experiencing symptoms such as breathlessness, cough, fatigue, and neurological symptoms (7); this condition was termed “post-COVID syndrome”. Symptoms may last from a few weeks to several months, severely impacting everyday life activities. In recent times, knowledge on post-COVID syndrome has increased dramatically, which explored clinical manifestations such as pulmonary, neurological, and thromboembolic complications (5, 8–11).

An alternative term called Long COVID (also referred to as “post-acute sequelae of COVID-19”) has recently evolved, which is characterized by a multisystemic condition ranging from severe to mild symptoms like severe acute respiratory syndrome coronavirus 2 (SARS-CoV-2) infection. Several reports show that at least 65 million individuals globally have long COVID (LC) (12, 13).

LC cases have been reported in all ages, and the severity level varies in different age groups; the age range 30–50 years has the highest percentage of diagnosis (14). It is characterized by multiple vital organs malfunction, including cardiovascular, thrombotic, and cerebrovascular disease (15), type 2 diabetes (16), and chronic forms of fatigue including myalgic encephalomyelitis/chronic fatigue syndrome (ME/CFS) and dysautonomia, especially postural orthostatic tachycardia syndrome (POTS) (17–20). The duration of these chronic symptoms can last from months to years, and in chronic conditions. The difference between COVID-19 and LC is based on the duration as well. The duration of ongoing COVID-19 is approximately 4–12 weeks, and if the COVID-19 symptoms last for more than 12 weeks, it is referred to as post-acute sequelae of COVID-19 (PASC) or LC (21, 22).

Several studies have formulated various hypotheses regarding the pathogenesis of LC such as persisting reservoirs of SARS-CoV-2 in tissues (23) after the COVID infection and immune dysregulation; pathogen reactivation, including herpesviruses such as Epstein–Barr virus (EBV) and human herpesvirus 6 (HHV-6), is another potential cause of LC (24). Other causes include the impact of SARS-CoV-2 on the set of naturally occurring microbiomes within the cells, blood clotting in micro vessels with endothelial dysfunction (25, 26), and dysfunctional signaling in the brainstem.

Multiple studies have reported that during the LC condition, the reactivation of EBV infection has been among the most reported phenomena. This reactivation process has also been associated with the severity of the COVID-19 illness (27, 28). Various longitudinal multi-omics studies have suggested that other risk factors such as type 2 diabetes, SARS-CoV-2 RNAemia, specific autoantibodies, and EBV viremia (29) associated with LC development. EBV infection causes various central nervous system illness conditions such as viral meningitis, encephalitis, sleep disorders, psychosis, and multiple sclerosis (30, 31). Previously, a study from Wuhan University (China) was carried out in which the population sample was co-infected with SARS-CoV-2 and EBV. The risk of severe symptoms was approximately threefold increased compared to the samples infected with SARS-CoV-2 only (32), indicating that EBV infection reactivation might directly contribute to increase in the severity of clinical symptoms.

Furthermore, Gold et al. (33) have reported that general symptoms of LC, such as fatigue, insomnia, headaches, myalgia, and confusion, might be because of the EBV reactivation by SARS-2CoV-2 infection. Another study that used COVID-19 patients (*n* = 309) has demonstrated that the EBV virus is greatly associated with LC symptoms (33). Taken together, the relationship between EBV reactivation and LC symptoms has been potentially established (33, 34).

Most studies have used just one-dimensional data, for example, either the observational data or the clinical/serological data, to prove their co-infection biological hypothesis, which is insufficient to provide a solid explanation for the pathogenic association mechanism in EBV-LC scenario and potentially associated genes (35–37). In the recent past, various scientists have applied next-generation sequencing (NGS) data especially the bulk RNA-Seq to decipher the association between two co-infections (38–41) and proposed the hub genes that could be used as biomarkers.

In this study, we have attempted to identify the common hub-genic profile between LC and EBV infection. We applied next-generation sequencing data, mainly RNA-Seq from the EBV-infected and LC-positive PBMCs combined with the protein structural bioinformatics data, and protein three-dimensional data using cutting-edge techniques such as molecular docking and dynamic simulations to explore the potential pathogenic correlation between EBV infection and LC at the genomic and protein structural level. We investigated the interaction of small molecules, specifically the bioflavonoids, with the shortlisted key hub genes to assess their potential as a drug candidate in mitigating the effects associated with LC and EBV infection scenarios. The results of this study will help us to understand the potential pathogenic connection and could serve as a tool to open a new therapeutics avenue against LC disease-associated manifestations.

## 2 Materials and methods

### 2.1 Data source

The bulk RNA-Seq from human peripheral blood mononuclear cells (PBMCs) of LC (42) 8^th^ month post-SARS-CoV-2 infection, non-LC, and the bulk transcriptome of 4^th^ day of post-EBV-infected PBMCs (43). The criteria of data selection were based on the patients with no treatment/wild samples. The high-resolution crystal protein structures of shortlisted drug target gene were downloaded from the RCSB Protein databank, and 3D structures of bioflavonoid small molecules were retrieved from PubChem (https://pubchem.ncbi.nlm.nih.gov/). The complete details of input data are given in **Supplementary Table S1**.

### 2.2 RNA-sequence data analysis

Sequence Reads Archives (SRA) files of RNA-Seq data from LC and EBV+ infection samples were converted into fastq raw format using fastq-dump and subjected to quality control analysis. The adapters, linkers, and overrepresented and poor-quality reads were evaluated and removed using TrimGlore (44). Refined raw reads were subjected to mapping against Human Genome 38 assembly (Hg38). STAR align (45) was used for the mapping at default parameters, and post-mapped files were formatted and sorted using SAMtools (46). The percentage of mapped reads are shown in **Supplementary Table S2**. It is crucial to count the number of mapped reads against the reference genome; we have used featureCounts (47), a part of the subreads platform, to quantify the mapped reads. FeatureCounts uses hg39_annotation.gtf bamfiles from normal and diseased samples as an input to quantify the reads at the gene level (47).

### 2.3 Identification of differentially expressed genes using DESeq2

After the mapped reads quantification, the next step is identifying the DEGs between conditions such as normal and diseased samples. The DESeq2 R-Package in R-studio package uses the shrinkage estimation for the read's dispersion and fold changes (FCs) among the sample; besides that, it also estimates the *p*-values and expression mean (48). First, the samples were normalized in a way that only the genes with greater than 10 reads counts were used for the DEGs analysis. We have selected two parameters, i.e., Log2FoldChange > 1 and *p*-values<0.05, to define the gene expression. The common DEGs (cDEGs) between LC and EBV+ infection samples were extracted using the “Venn” R-package. The final output files were exported in the excel format.

### 2.4 Gene ontology analyses

Significant cDEGs were subjected to identify functional categories such as gene ontology (GO), including biological process (BP), cellular component (CC), and molecular functions (MF), and underlying pathways like Kyoto Encyclopedia of Genes and Genomes (KEGG) pathway enrichments in the web-based platform ShinyGo 0.80 version (49) (http://bioinformatics.sdstate.edu/go/) that uses the list of DEGs as an input. To ensure the quality of results, we used false discovery rate (FDR) values > 0.05 as a threshold and the top 20 enrichment terms were visualized.

### 2.5 Protein–protein interaction network analysis

Cells perform various complex functions mediated by the regulatory interactions among several proteins. Therefore, to get an insight into the involvement of common genes in PPIs, we used the STRING database (https://string-db.org/). This database collects data from different sources via text-mining from published literature, computational predictions using co-expression information, and conserved genomic landscape of genes (50). We have used official symbols of common genes as an input to predict their PPI network. PPIs with an interaction score > 0.4 were refined and used for downstream steps.

### 2.6 Hub gene identification and transcription factor enrichment analysis

In the genome, some genes are involved in the formation of frequent interactions with other genes; such genes are called hub genes. These genes are essentially involved in the formation of a regulatory network and play a crucial role in gene regulation and biological processes (51). Hub genes were detected using the CytoHubba plug-in followed by conformation through different modules (degree) (52). CytoHubba takes PPI network as an input; therefore, the output of STRING was exported and visualized in cytoscape's plug-in CytoHubba (53). Molecular Complex Detection (MCODE) (degree cutoff = 2, max depth = 100, node score cutoff = 0.2 and K-core = 2) was applied for the clustering analysis (54) followed by the ClueGo to carry out KEGG and GO analysis (55). Since gene expression is mainly controlled by transcription factors (TFs), it is thus worthy to perform a TF analysis for the hub genes to get an insight into the correlation between hub gene expression and TF enrichment. Therefore, we used the EnrichR (56) platform to identify the TF-lof enrichment and ENCODE TF ChIP-Seq enrichment.

### 2.7 Molecular docking

Molecular docking is an efficient method to identify the interactions between proteins and small molecules. The three-dimensional protein structure's selected hub gene OLR1 (PDB ID: 7XMP) was retrieved from the RCSB protein data bank (https://www.rcsb.org/) in pdb format. The attached ions, solvent, and unsaturated molecules were removed using the Chimera visualization tool (57); protein was prepared using Chimera's Dock Prep function, and hydrogen and other missing atoms were added and subjected to molecular docking using the *PyRx* software (58). The small bioflavonoids molecules library of bioflavonoids was downloaded from PubChem database (https://pubchem.ncbi.nlm.nih.gov/) and converted into the pdb format. The AutoDock suite was applied, the small molecules were imported as a library, their energies were minimized using *uff force field*, and conjugate gradients were used as the optimization algorithm saved in *pdbqt* format. The maximize grid was selected and blind docking was performed. The best docking pose was selected based on the lowest binding energy and root mean square deviation (RMSD) value. The two-dimensional view of interacting residues was visualized using Discovery Studio (59).

### 2.8 Molecular dynamics simulations of receptor–ligand complexes

MD simulations were conducted using AMBER (60) and GROMACS software suites (61) with the Amber force field (62). Initial coordinates and topology files of both receptor and ligands were generated by using Gaff force field 2 and ff99SB force field, respectively. Both ligands and proteins were prepared via the Antechamber and the Leap program of Amber tools. The particle mesh Ewald method was applied to compute the long-range electrostatic interactions while the short-range interactions, such as van der Waals interactions, were calculated with a default cutoff value (1.0 nm). The system was solvated using the OPC water model with a size of 12.0 Å. The GROMACS.top and.gro files were generated for the MD simulations run using the amb2gro_top_gro.py tool, which were based on the AMBER prmtop and inpcrd files (60). Energy minimization was performed on the receptor–ligand complexes and unbound proteins using the GROMACS v. 2021.4-plumed-2.8.0 software package. The initial 2,000 steps involved minimizing steepest descents, while the subsequent 2,000 steps were for minimizing the conjugate gradients. The temperature equilibration was performed at 300 K temperatures for 50,000 steps. After the equilibration step, the prepared systems of both apo and ligand-bound complexes were subjected MD simulations for 100 ns at 300 K temperature and 1 atm pressure. Several downstream analyses such as RMSDs, radius of gyration (Rg), root mean square fluctuations (RMSFs), hydrogen bonding analysis, and energy decomposition were carried out. The Bio3D (63) r-package was used to calculate the principal component analysis (PCA) and the dynamic cross-correlation map (DCCM). The analysis output graphs were plotted using the r-package ggplot2. Molecular mechanics Poisson–Boltzmann surface area (MMPBSA) is the remarkable method used to calculate the binding free energy (in kcal/mol) of a complex between protein and small molecules (64). The determination of the binding free energy denoted as Δ*G*_bind_ between a ligand and a protein in the formation of a complex in aqueous solution is based on the disparity stated as follows:


(1) $$\Delta G_{bind}=G_{complex}-\left(G_{Protein}-G_{Ligand}\right)$$

(2) $$\Delta G_{bind}=\;\Delta G_{gas}+\Delta G_{sol}+T\Delta S$$

(3) $$\Delta G_{gas}=\;Bond+Angle+Dihed+EEL+VDWAAL$$

(4) $$\Delta G_{sol}=\Delta EGB+\Delta ESURF$$

## 3 Results

### 3.1 Mapping and analysis of differentially expressed genes

**Figure 1:**
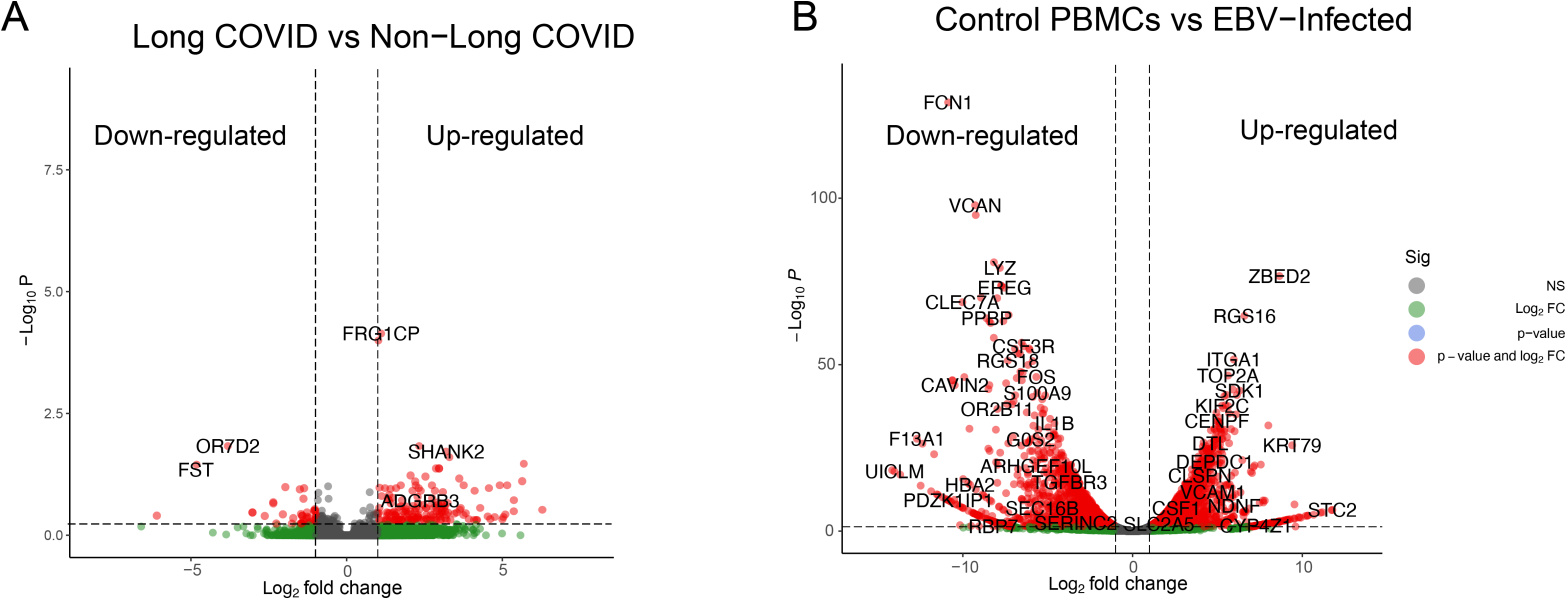
Identification of differentially expressed genes (DEGs) in LC and EBV-infected RNA-Seq data. Up and down regulated genes are represented by a single dot. **(A)** DEGs from 8^th^ month post-SARS-CoV-2-infected PBMCs (42). **(B)** DEGs from 4^th^ day post-EBV-infected PBMC samples (43). Log2FC values are plotted on the *x*-axis and −Log10 *p*-values are shown on the *y*-axis. Green dots represent the genes that are greater than log2FC>1, gray dots are non-significant genes, red dots show the genes that fulfill the criteria of *p*-values< 0.05 and log2FC collectively, and blue dots represent genes with *p*-value<0.05 only.

The processed clean raw reads were mapped against the human genome reference; mapping reads percentage statistics are presented in **Supplementary Table S2**. The mapped output files are saved in bam files format, the low-quality mapped reads were filtered out with the “-bq1” parameter, and the resulting files were sorted and saved again in new bam files. Filtration and sorting were performed using SAMtools (46). In LC data, nearly ~22,500 genes were found to be expressed. In order to differentiate between the up- and downregulated genes, we used the following criteria: log2FC > 1 and log2FC< −1, and *p*-value<0.05, respectively. Out of ~22,500 expressed genes, 866 genes were found to be upregulated and 330 genes were found to be downregulated while rest of the genes did not meet the criteria of significance and considered as outliers. In EBV samples, a total of ~19,700 genes were detected as expressed genes, and similar to LC samples, up- and downregulated genes were detected in EBV data with the following criteria: log2FC > 1, log2FC< −1, and *p*-value<0.05, respectively. A total of 3,075 genes fulfilled the upregulation criteria of DEGs, where 2,806 genes were found to be downregulated. The rest of the genes did not fulfill the criteria specified and were considered as non-significant. The genome-wide upregulated and downregulated genes of both LC and EBV-infected samples are shown in **Figure 1**. Collectively, we found that in LC and EBV-infected samples, several genes were dysregulated. Detection of gene expression within the samples is important because it enables us to get more explicit details about the expression pattern of each replicate. Therefore, we selected the top genes from the genome-wide mRNA samples and plotted them in an expression heatmap (**Supplementary Figure S1**). The selection of genes was based on the log2FC and *p*-values, together. Among the top 30 genes, 25 genes were found to be upregulated in non-LC samples while 5 genes [FST (65), OR7D2 (66), SERPINE1 (67), TMEM176A (68), and TMEM176B (69)] were dysregulated in the LC sample cluster. The association of these five genes with LC/COVID-19 has been previously reported by various scientists via bulk RNA-Seq and genomic data analysis. A clinical genomic data study has reported that follistatin (FST), which is an inhibitor of the follicle-stimulating hormone (FSH), has been upregulated (36).

In EBV samples, the top 20 genes were plotted, and 18 genes were found to be downregulated on the 4th day of post-EBV infection samples, while two genes ZBED2 and RGS16 were among the upregulated ones in EBV-infected samples.

### 3.2 Protein–protein interaction analysis of common genes and identification of hub genes

**Figure 2:**
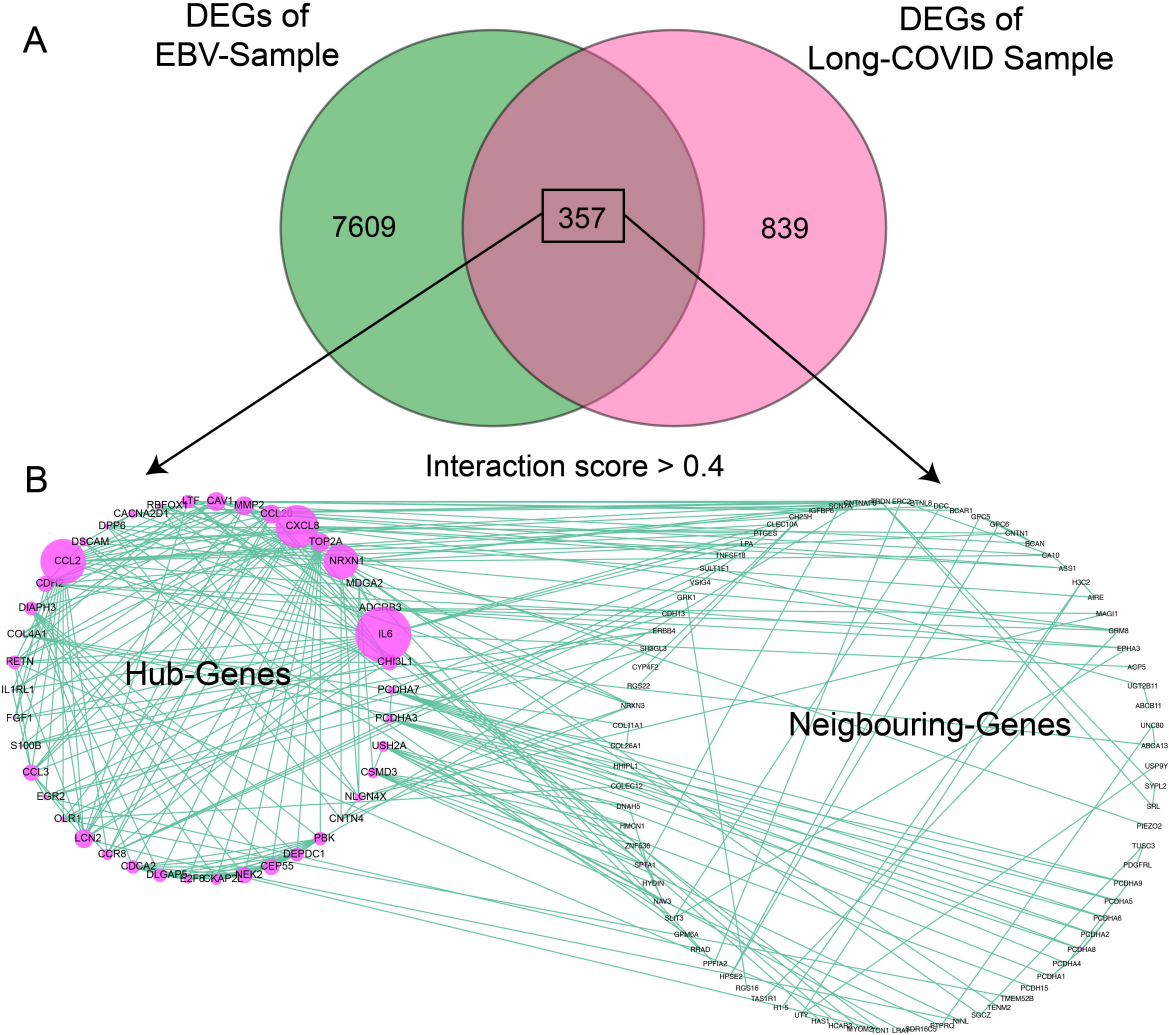
Identification of common DEGs between LC and EBV-infected samples and PPI analysis. **(A)** Venn diagram showing the common genes between LC and EBV samples. **(B)** The circos plot represents the PPI networks of common genes having an interaction score > 0.4 (*medium interaction score*) created by the STRING database. The left panel shows the hub genes predicted by the *Cytohubba* plugin and the right panel indicates the neighboring genes or non-hub genes. Each green line shows one string and purple circles show the nodes.

**Figure 3:**
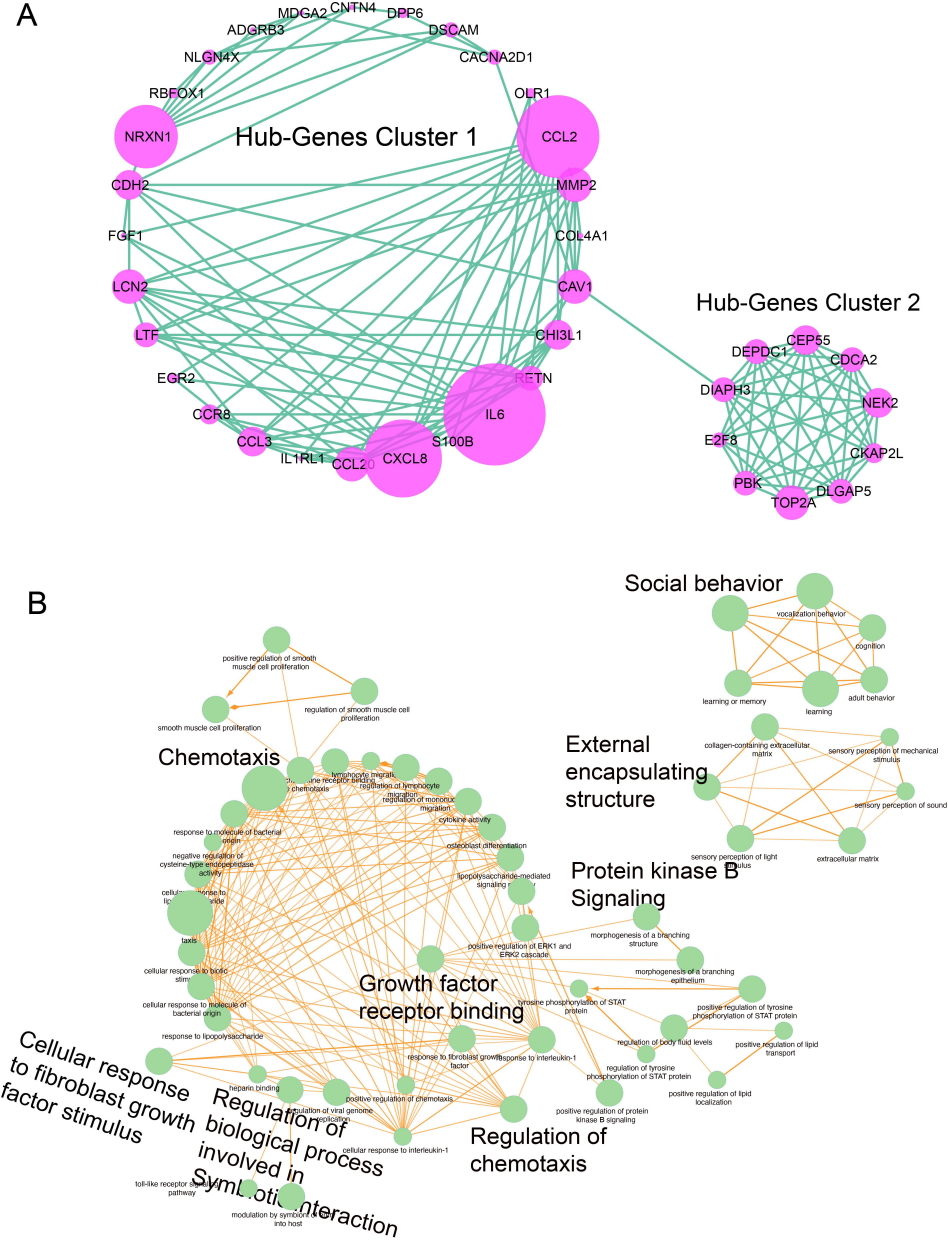
Hub genes clustering based on the number of nodes and enrichment analysis. **(A)** Clustering analysis was performed by applying different modules in MCODE, (degree of connection) was applied, and two clusters were detected. The size of a ball indicates the number of associated nodes (smallest degree = 5); a higher number has a bigger size (highest degree = 25). Each green line show one string and purple circles show the nodes. **(B)** Enrichment analysis of hub genes, in which each green ball represents the hub gene and its involvement in various biological functions; significant terms were assessed using adj. *p*-value<0.05. The size of the node indicates the significance of GO terms. The yellow line shows the nodes interacting with each other.

Significant DEGs from both samples (7,609 genes from EBV samples and 839 genes from LC samples) were intersected; 357 common/overlapped DEGs between two samples were extracted and considered as the common signature genes between both diseases. The intersection criteria were based on the gene names and were plotted in a Venn diagram as shown in **Figure 2A**. The enrichment analysis of the cDEGs showed that only five genes were functionally enriched in glutathione transferase activity with an FDR of 4.1E-03 and 24 genes were enriched in calcium ions binding with an FDR of 1.5E-03. The common genes were subjected to PPI analysis using the STRING website. The network with the interaction score > 0.4 from the STRING server was exported to cytoscape's plugin CytoHubba to identify hub and neighboring genes. A total of 125 genes were involved in the formation of PPIs (**Supplementary Figure S2**). Out of these 125 genes, 43 were classified as hub genes [with more frequent edges (degree > 5) and strings] predicted by CytoHubba using multiple built-in modules and are shown in **Supplementary Table S4**, while 82 genes were classified as neighboring genes as shown in **Figure 2B**. The hub genes were further broken down into clusters via the MCODE plugin. Out of the 43 hub genes, 28 were clustered together in cluster 1, while the rest of the genes were clustered together in cluster 2 as shown in **Figure 3**. In cluster 1, CCL2, MMP2, IL6, CXCL8, CCL20, NRCN1, and CDH2, had the highest number of edges. In cluster 2, NEK2, TOP2A, CDCA2, CEP55, DEPDC1, DIAPH3, PBK, and DLGAP5 had the highest number of edges. These genes were highlighted with a purple bubble as presented in **Figure 3A**. Since these genes have an ability to interact frequently, it is worthy to study in detail their role in generating any pathogenic association between LC and EBV reactivation.

We were interested to determine their role in biological pathways. Most of the genes exhibit their biological involvement in the pathways shared by LC and EBV infection, for example, the behavioral maintenance-related pathways. Hub genes were subjected to GO analysis; functional analysis, including biological process, cellular components, and molecular functions, and KEGG pathway analysis were performed as plotted in **Figure 3B**. It was found that the hub genes were involved in the following GO terms: (1) growth factor receptor binding, (2) maintain external encapsulating structure, (3) protein kinase B signaling, (4) social behavior associated, (5) regulation of chemotaxis, (6) regulation of biological processes involved in symbiotic interactions, and (7) cellular response to fibroblast growth factor stimulus.

### 3.3 Differential expression of hub genes in LC and EBV and analysis of transcription factors

Since hub genes have gained a great functional importance, we have identified their individual gene expression in both LC and EBV samples. We chose the list of genes with frequent edges and investigated their individual gene expression in both LC and EBV samples. We selected the genes with dense edges (degree > 5) and identified their gene expression in both conditions. We have found that CDCA2, CEP55, CKAP2L, DEPDC1, DIAPH3, DLGAP5, E2F8, FGF1, NEK2, PBK, and TOP2A were upregulated in both LC and EBV infection. Only CCL2, CHI3L1, and EGR2 showed the opposite expression, i.e., higher gene expression in EBV sample and lower gene expression in LC. The expression of MMP2 and RETN was upregulated in LC- and downregulated in EBV+ infection samples.

**Figure 4:**
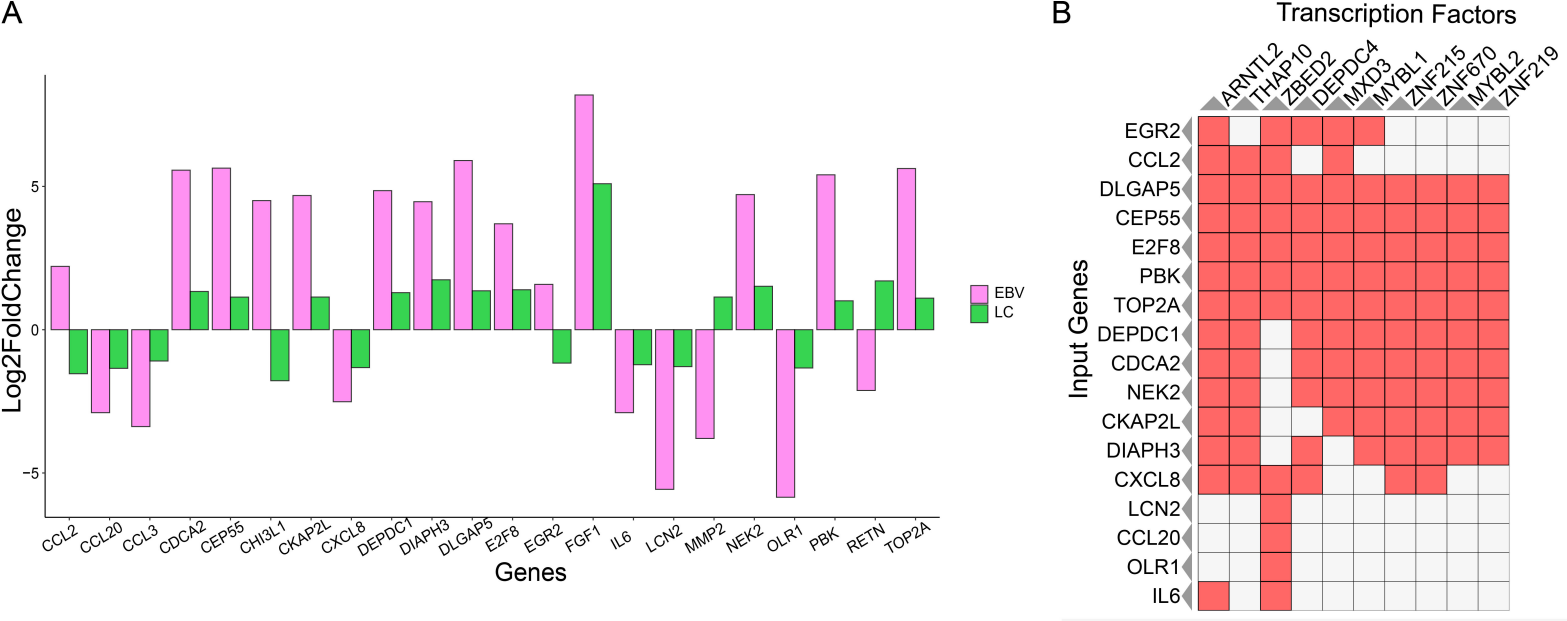
Individual gene expression and TF analysis. **(A)** Hub gene expression in EBV and LC samples. Pink represents EBV infection samples, and forest green indicates the LC samples. **(B)** TF-enrichment analysis for the hub genes. The red box represents the presence of TF, and the white box represents the absence of TF.

On the other hand, CCL20, CCL3, CXCL8, DEPDC1, DIAPH3, DLGAP5, E2F8, IL6, LCN2, and OLR1 were depleted in both EBV+ infection samples and LC data. The expression was calculated on the basis of FC value. The range that we observed was from −6 to +6. In EBV infection, CDCA2, CEP55, CKAP2L, DEPDC1, DIAPH3, DLGAP5, FGF1, PBK, TOP2A, CCL3, LCN2, MMP2, OLR1, IL6, and LCN2 were the genes exhibiting a significant change of expression (based on log2FC) as compared to others. However, in LC samples, only the FGF1 gene was observed to produce a higher log2FC as shown in **Figure 4A**. The gene expression is controlled by the TFs.

Next, we performed the TF enrichment analysis in selected hub genes. It was found that out of 22 genes, only 17 were enriched with multiple TFs and most of the genes shared a common TF. For example, the TF ARNTL2 controlled multiple genes such as EGR2, CCL2, DLGAP5, CEP55, E2F8, PBK, TOP2A, DEPDC1, CDCA2, NEK2, CKAP2L, DIAPH3, CXCL8, and IL8. Additionally, we have found that TFs belonging to the Zinc-finger protein family and MYB transcription family were among those associated with the 10 hub genes. Previous studies have also shown the association of these family protein with COVID-19's manifestations and EBV infection as shown in **Figure 4B**. DLGAP5, CEP55, E2F8, PBK, TOP2A, DEPDC1, CDCA2, NEK2, CKAP2L, and DIAPH3 were reported as the genes associated with the MYB and ZNF-TF families. Three members of the ZNF-TF family, i.e., ZNF-215, ZNF-2670, and ZNF-219, were predicted as the main TFs controlling the hub genes. In the MYB family, MYBL1 and MYBL2 were the members identified as the TFs showing the regulatory association with the hub genes. Since hub genes are the key genes that are involved in controlling various biological functions, it is essential to monitor their gene expression. We identify the expression of hub genes and found that their expression changes depending on EBV and LC conditions.

Furthermore, we identify the TFs involved in controlling these hub genes; common TFs are shared by the genes that co-regulate in EBV or LC, suggesting that these genes shared a common transcriptional machinery. For example, the TF ARNTL2 is associated with 14 out of 17 hub genes in the list (**Figure 4B**), and it was observed that these genes were either upregulated or downregulated together in EBV and LC.

### 3.4 Molecular docking with bioflavonoids

**Figure 5:**
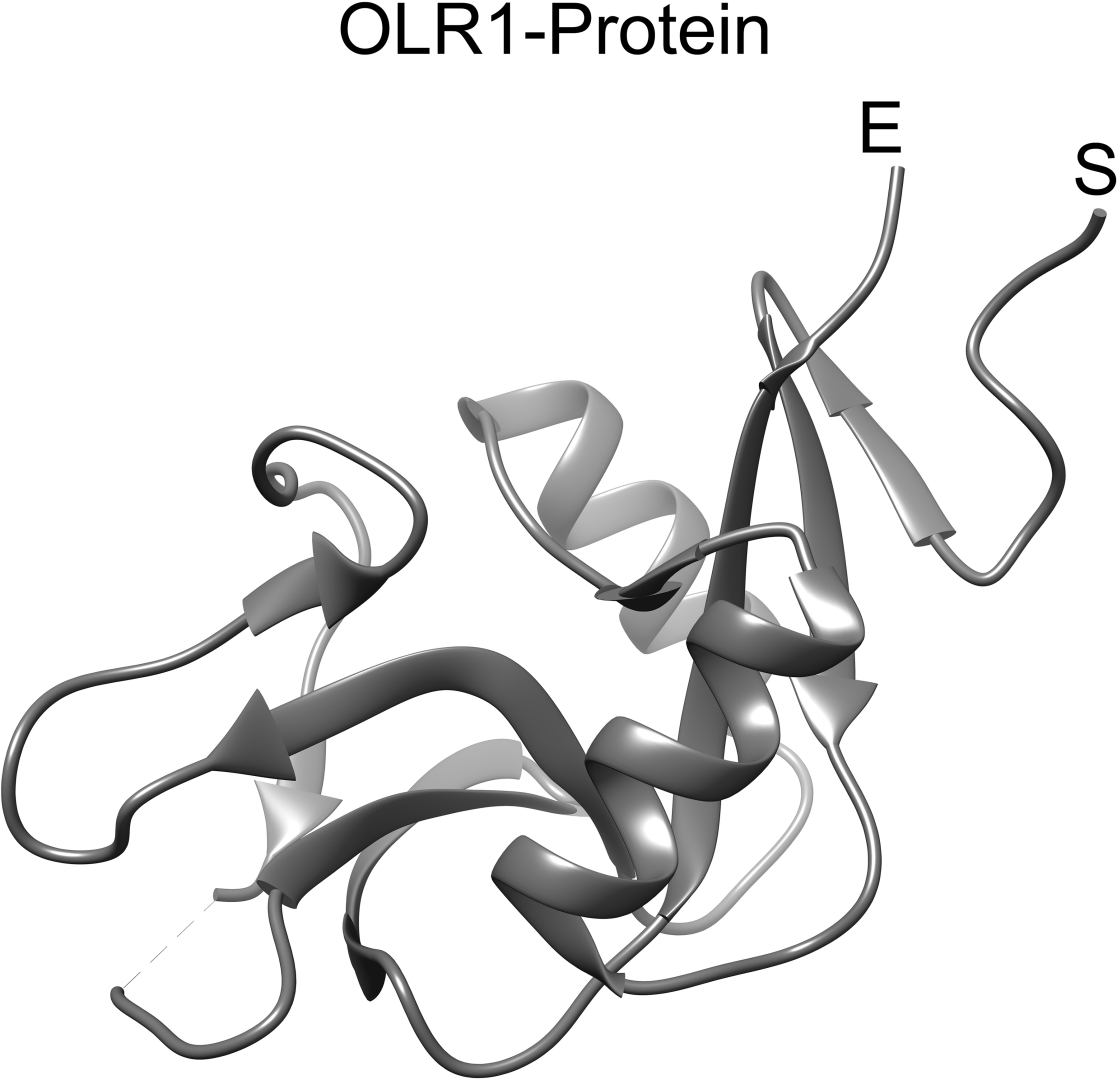
OLR1 protein structure. The 3D structure of OLR1 gene encoded protein (PDB ID: 7XMP) was retrieved from RCSB PDB. The ribbon representation of OLR1 is visualized in UCSF chimera. The start and end of the protein structure is labeled “S” and “E”, respectively.

**Figure 6:**
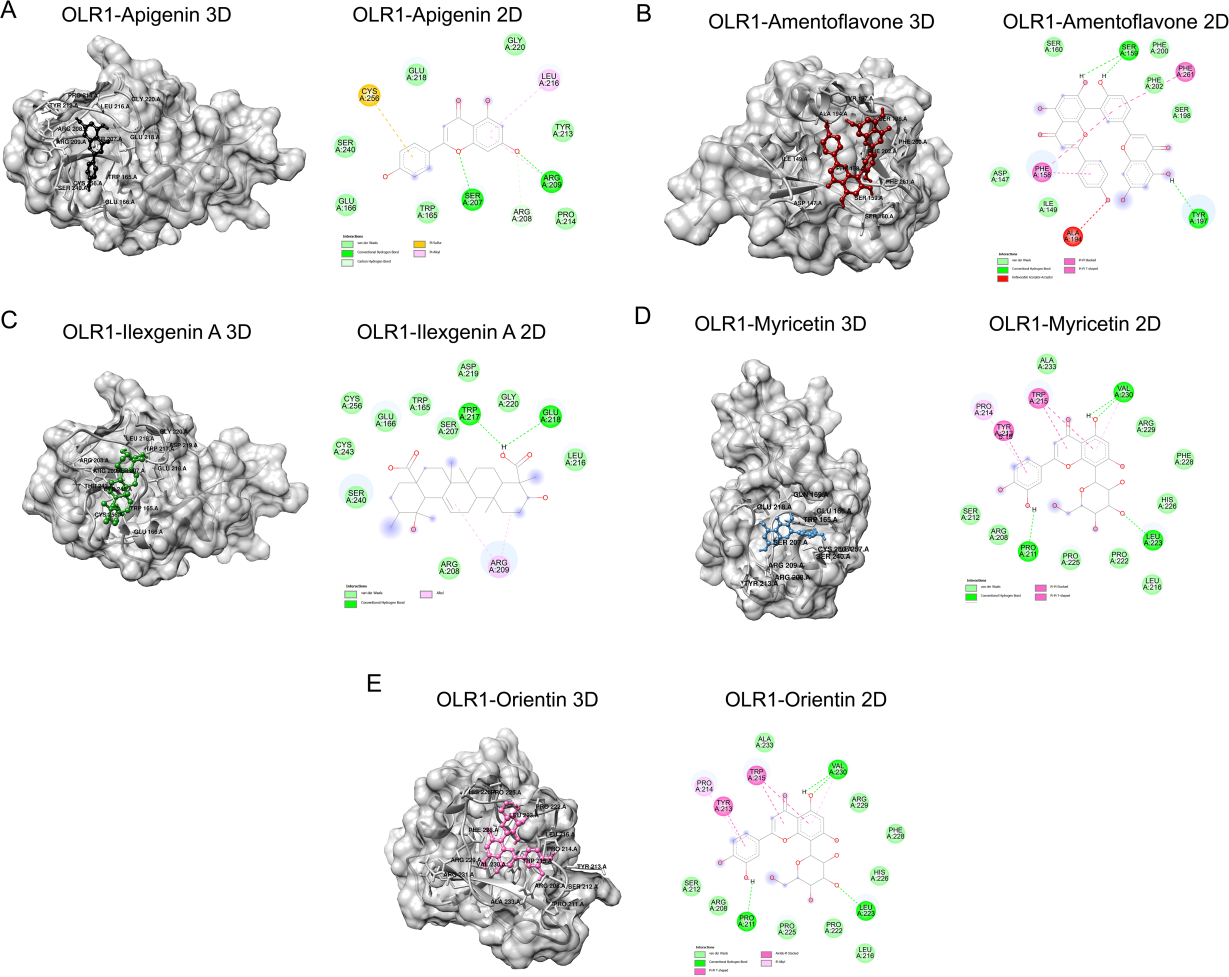
Binding analysis of OLR1 protein and small molecules. **(A)** The surface view of docked complex (left panel) between Apigenin and OLR1 protein (PDB ID: 7XMP). The ligand is shown in black and the receptor protein is shown in gray. **(B)** Amentoflavone and OLR1 protein. The ligand is shown in deep red. **(C)** The surface view of the docked complex (left panel) between Ilexgenin A and OLR1 protein. The ligand is shown in deep green. **(D)** Myricetin and OLR1 protein. The ligand is shown in light blue. **(E)** Orientin and OLR1 protein. The ligand is shown in pink. The bound amino acids are labeled in black. Two-dimensional representation of docked residues is shown in the right panel. The drug molecules are shown in “stick” representation. The bound residues are shown in different colors, each color representing different types of interactions. The dotted lines represent the bonds.

In this study, we have shortlisted the hub genes that have a direct association with SARS-CoV-2 infection-related diseases and EBV infection. The selection criteria were solely based on the literature survey. We have used Lectin-like Ox-LDL receptor 1 (OLR1) and searched their corresponding proteins (PDB ID: 7XMP) from PDB with 1.27 Å resolution (**Figure 5**). The protein was docked against biological active compounds mainly in the bioflavonoid library. It was previously reported that bioflavonoids have a strong antiviral and antitoxic ability (70). The top five drug/compounds were used for further analysis (**Supplementary Figure S3**). In the OLR1–apigenin complex, the binding energy was −7.6 kcal/mol, and Ser207 and Arg209 mainly formed hydrogen bonds with apigenin (**Figure 6A**). Amentoflavone docked with OLR1 protein and showed a binding energy of −8.3 kcal/mol. Two amino acids, Phe158 and Phe261, were involved in pi bonds, while Ser159 and Tyr179 formed conventional hydrogen bonds as shown in **Figure 6B**. In the OLR1–ilexgenin A complex, the binding energy was −7.6 kcal/mol. A total of 13 amino acids were involved in the interaction formation. Out of these 13 bonds, two residues, i.e., Trp217 and Glu218, formed hydrogen bonds as represented in **Figure 6C**. In the OLR1–myricetin complex, the binding energy was −6.5 kcal/mol and a total of 16 amino acids were involved in the interaction formation. Out of these 16 bonds, three amino acid residues, i.e., Val230, Leu223, and Pro211, formed hydrogen bonds and the three-dimensional view and bound residues are shown in **Figure 6D**. In the OLR1–orientin complex, the binding energy between ligand and receptor was −7 kcal/mol. A total of 15 amino acids were involved in the interaction formation. Three residues, i.e., Pro211, Val230 and Leu223, formed hydrogen bonds, and three amino acids, Tyr213, Pro214, and Trp215, formed the pi bonds. Other residues such as Ser212, Arg208, Pro225, Pro222, Leu216, His226, Phe228, and Arg229 were bound with orientin via van der Waals interactions as presented in **Figure 6E**. Overall, a variety of bonding was observed, such as hydrogen bonding, van der Waals interactions, sulfur interactions, pi bonds, and C–H bonding; a detailed list of bonding is shown in **Table 1**.

**Table 1:** Docking results for lectin like Ox-LDL receptor 1 (PDB ID: 7XMP).

Sr. no.	Compound (Pubchem CID)	Binding affinity (kcal/mol)	Interactions at the binding site		
			H-bond interaction	Van der Waals interactions	Other interactions
**1**	Apigenin (5280443)	−7.6	Ser207, Arg209	Ser240, Glu166, Trp165, Arg208, Pro214, Tyr213, Gly220, Glu218	Cys256, Leu216
**2**	Amentoflavone (5281600)	−8.3	Ser159, Tyr197	Asp147, Ile149, Ser198, Phe202, Phe200, Ser160	Phe261, Phe158, Ala194
**3**	Ilexgenin A (21672638)	−7.6	Trp217, Glu218	Arg208, Leu216, Glu218, Gly220, Asp219, Trp165, Glu166, Cys256, Cys243, Ser240	Arg209
**4**	Myricetin (5281672)	−6.5	Val230, Leu223 Pro211	Ala233, Arg229, Phe228, His226, Leu216, Pro222, Pro225, Arg208, Ser212	Trp215, Tyr212, Pro214
**5**	Orientin (5281675)	−7	Pro211, Leu223, Val230	Ser212, Arg208, Pro225, Pro222, Leu216, His226, Phe228, Arg229, Ala233	Trp215, Tyr212, Pro214

### 3.5 Molecular dynamics simulations analysis of OLR1 protein and complexes

The molecular changes and stability of small-molecule inhibitors–OLR1 complexes were investigated from their corresponding 100-ns MD simulation trajectories. The trajectories obtained from 100-ns simulations for the hub gene protein complexed with apigenin, amentoflavone, ilexgenin A, myricetin, and orientin systems were prepared using the Antechamber of the Amber program (62). The atom-positional RMSD generated by roto-translational least-squares fitting is perhaps the most widely used for structural comparison and stability measure.

**Figure 7:**
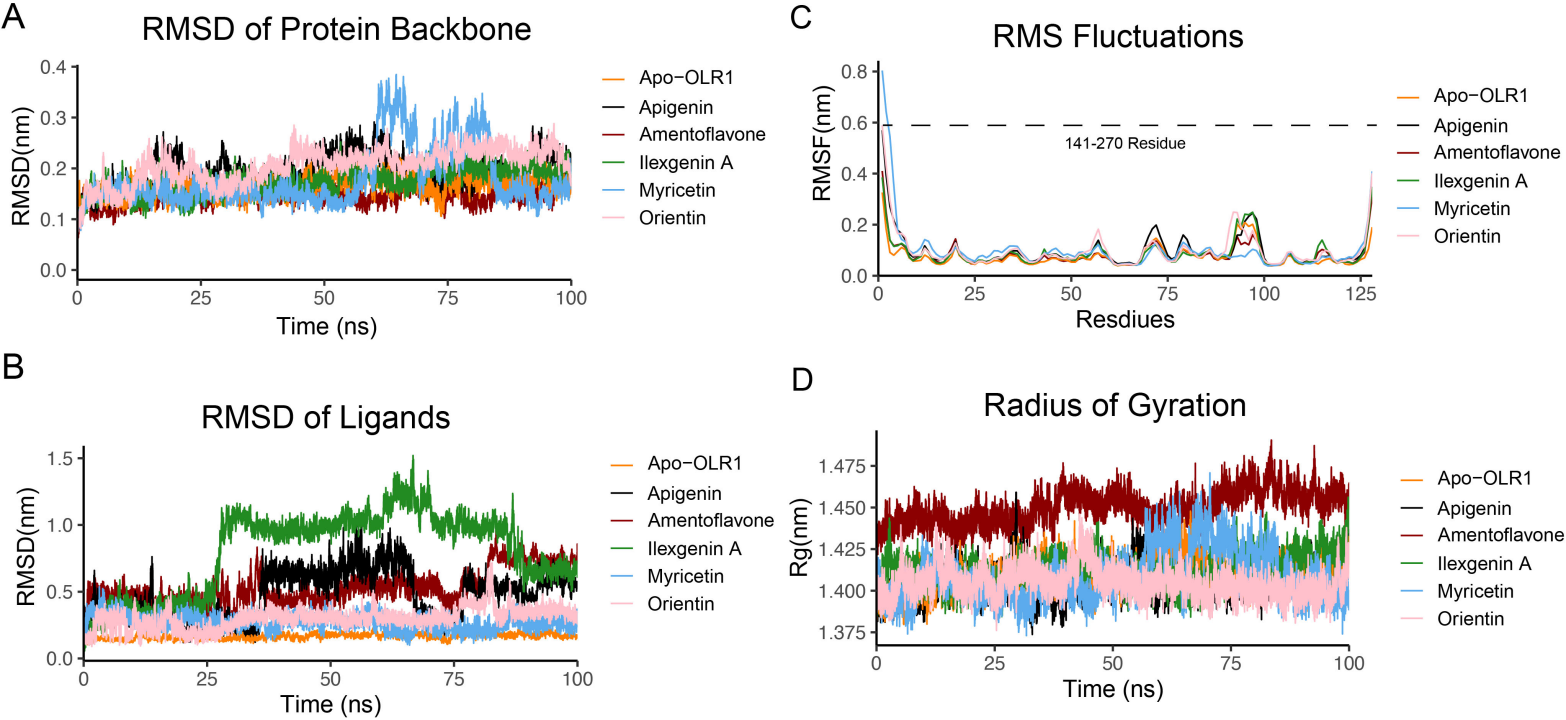
Protein and ligand complex stability analysis. **(A)** RMSD of OLR1 protein backbone, **(B)** RMSD of bound ligands. The *y*-axis shows the fluctuation values in nanometers (nm), and the *x*-axis represents the simulation time frames in nanoseconds (ns). **(C)** RMSF of protein backbone, the *x*-axis represents the total number of residues, which are 127 (141–270). **(D)** Radius of gyration. The *y*-axis shows the fluctuation values in nanometers (nm), and the *x*-axis represents the simulation time frames in nanoseconds (ns). Each ligand is represented by the different colors.

We analyzed the structure stability of the backbone of protein in the complexes and in unbound form. The structure remained intact throughout the simulation time of 100 ns (**Figure 7**). RMSD plots of OLR1 complexed with the five ligands showed the fluctuations ranging between ~0.1 and 0.4 Å. All the five ligand-bound complexes up to 100 ns remain intact. However, the protein bound with myricetin showed some fluctuation from ~60 to 70 ns comparatively. Later, another fluctuation was observed at 75 to 80 ns followed by a stable conformation. After myricetin, orientin showed slight fluctuations; overall, the complexes exhibit the compactness of a complex structure. From the RMSD analysis of ligands, all ligands remained intact up to 25 ns, and no motion was observed, except for the ilexgenin A, which showed larger fluctuations in RMSD values, from 0.5 to 1 nm. It remained steady at the same value until 80 ns. It was also observed that the ligands' moment comes down to nearly ~0.6 nm and then remained steady until 100 ns. After ilexgenin A, the second ligand apigenin showed changes in ligand moment from ~30 to 60 ns. It remained steady until the end of simulation. The third ligand amentoflavone showed changes in ligand moment from 45 to 65 ns. Overall, the fluctuation rate was from 0.0 to 1.5 nm, while the other ligands did not exhibit any significant changes in moment (**Figures 7A, B**).

The RMSF analysis, as depicted by the RMSF graph (**Figure 7C**), elucidates the dynamic characteristics of specific regions within a protein structure. The RMSF graph shows that amino acid residues of the protein exhibit a higher degree of movement or flexibility. Quantitatively, these fluctuations were observed to extend up to 0.8 nm. Overall, the fluctuations reached 0.2 nm. Further inspection of the RMSF data reveals that the residues constituting the active site, as well as those in its vicinity, demonstrate considerable stability, with an average RMSF value of approximately 0.17 nm. Additionally, the analysis highlights a specific loop region, spanning from His226 to Cys243, exhibiting fluctuations up to 0.3 nm.

All five complexes showed a similar pattern in terms of Rg value. A noteworthy observation from the analysis is that all five complexes exhibited a consistent pattern regarding their Rg values (**Figure 7D**). This consistency in Rg values across the different complexes suggests a level of long-term stability and compactness within their structures, indicating that, despite the dynamic environment of the MD simulations, these complexes maintain their structural integrity over time. Specifically, the myricetin complex was identified to exhibit notable fluctuations from 60 to 80 ns within the MD simulation trajectory.

**Figure 8:**
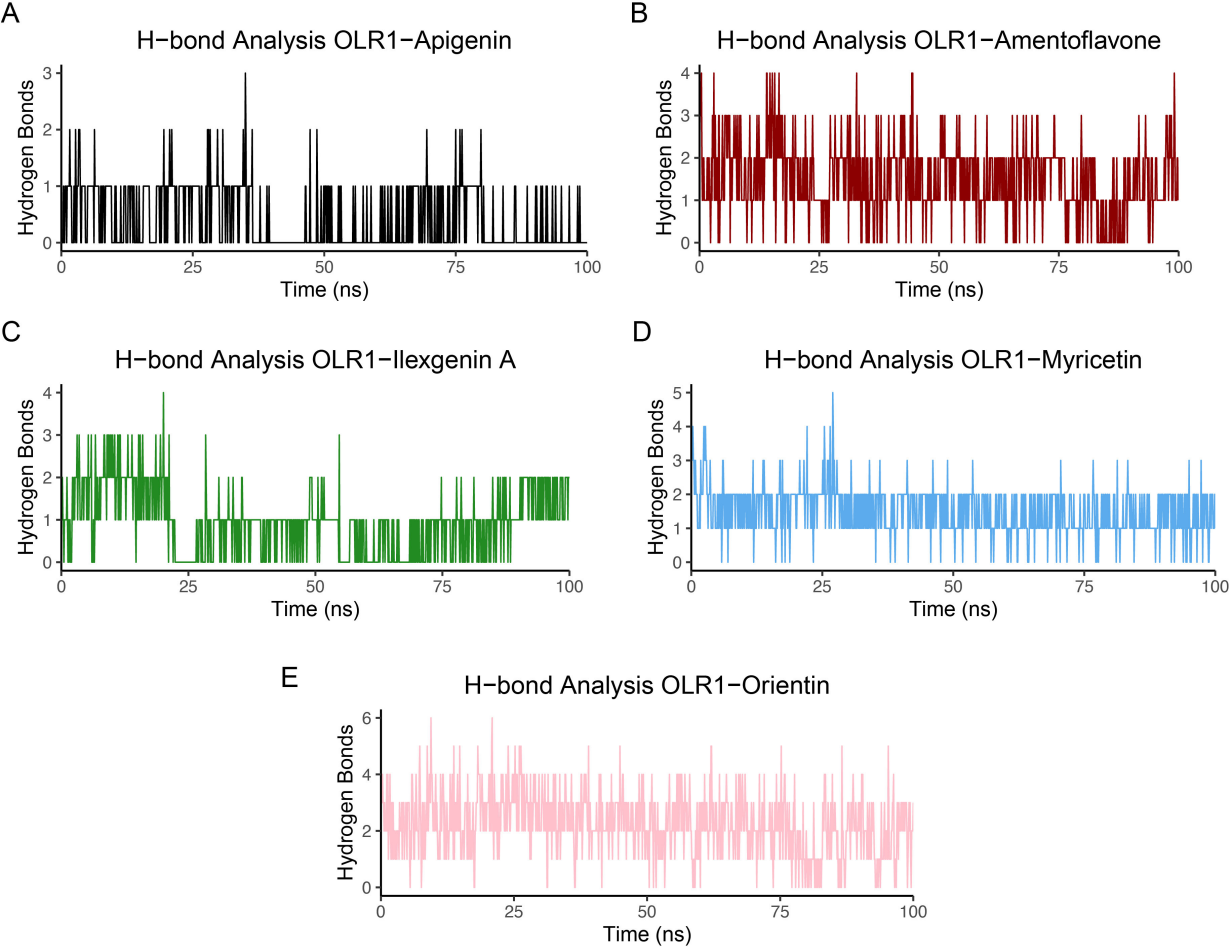
Hydrogen bond analysis between OLR1-receptor and bioflavonoid molecules. **(A)** OLR1–Apigenin, **(B)** OLR1–Amentoflavone, **(C)** OLR1–Ilexgenin A, **(D)** OLR1–Myricetin, and **(E)** OLR1–Orientin. The time frames of MD are plotted on the *x*-axis, and the number of hydrogen bonds is shown on the *y*-axis.

The number of hydrogen bonds formed between ligands and protein target is shown in **Figure 8**. Myricetin and orientin formed high average numbers of hydrogen bonds compared to the other ligands. Myricetin formed five hydrogen bonds and orientin formed six hydrogen bonds with OLR1, whereas apigenin, amentoflavone, and ilexgenin displayed three, four, and four average hydrogen bonds, respectively. The average amount of hydrogen bonds produced over the whole simulation time may be helpful in determining which molecule is continually producing the greatest number of hydrogen bonds. Furthermore, we have identified the individual residues involved in the hydrogen bond formation across 100 frames. The residues mainly involved in two kinds of hydrogen bonding, such as bonding between backbone and side chain or bonding between side chain and side chain, were observed and are shown in **Supplementary Figure S4**. Among all the complexes, the number of residues involved in the hydrogen formation varies based on the attached ligands. Additionally, the lifetime of these hydrogen bonds was also examined, with specific criteria for analysis, including a cutoff radius of 0.35 nm and an angle cutoff of 120° (71).

**Figure 9:**
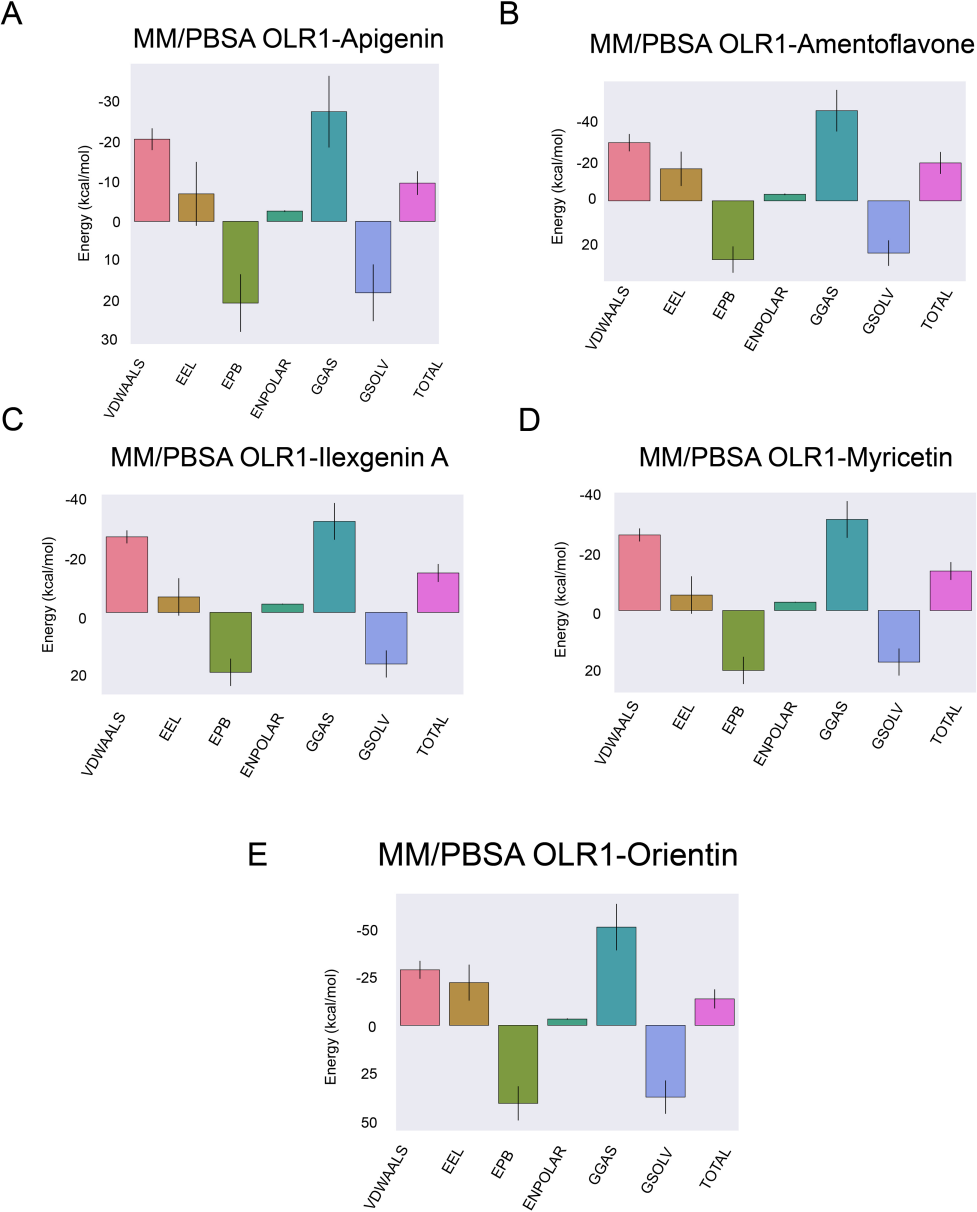
Binding free energy calculations. Energy calculations were performed using the MMPBSA method for each ligand and receptor complex. **(A)** OLR1–Apigenin, **(B)** OLR1–Amentoflavone, **(C)** OLR1–Ilexgenin A, **(D)** OLR1–Myricetin, and **(E)** OLR1–Oreintin. The *x*-axis indicates the energy level in kcal/mol and types of energies are plotted on the *y*-axis.

The MMPBSA net binding energy order from the highest to lowest stability was as follows: amentoflavone −18.48 kcal/mol, myricetin −14.13 kcal/mol, orientin −13.75 kcal/mol, ilexgenin A −11.07 kcal/mol, and apigenin −9.55 kcal/mol (**Figure 9**). Among all five complexes, amentoflavone, myricetin, and orientin had minimum binding energy, indicating these compounds as effective inhibitors (72). The lower binding energies for these complexes suggest that they have a higher potential to effectively inhibit the target (73), making them significant candidates for further investigation in drug discovery efforts against the OLR1. The complete details of binding energies are given in **Supplementary Table S3**.

PCA was applied to calculate the motion of OLR1 protein in the bound and unbound state with the ligands (63). Additionally, the correlations between the conserved region of the protein were determined by computing a dynamic cross-correlation matrix (DCCM) (63) (**Supplementary Figure S5**). The PC1, PC2, PC3, and eigenvalues of apo were plotted against the respective eigenvector index for the first 20 modes of motion. Overall protein movement was controlled by eigenvectors, especially the higher ones, and the top five eigenvectors in our system demonstrated dominant movements with eigenvalues of 16.6%–60.6% while the remaining eigenvectors had lower eigenvalues. According to the PCA plot, the PC1 cluster retained the highest variability of 16.62%, PC2 exhibited 9.75% variability, while PC3 showed minimal variability (8.67%) (**Supplementary Figure S6**).

## 4 Discussion

The pathogenic association between EBV infection and LC has been previously reported by various studies (28) using clinical findings. It is believed that LC occurs as the result of the SARS-COV-mediated EBV reactivation mainly in the COVID-19 recovered population (74). However, their genomic bases of pathogenic correlation need to be deeply explored; moreover, it is imperative to identify the key genes and their role in molecular pathways that could possibly act as biomarkers under LC conditions. The significance of association between COVID and EBV has been previously heightened by the studies of hospitalized COVID patients showing that reactivated EBV significantly increased mortality when compared to EBV-negative patients (75, 76). Recently, apart from the molecular techniques, NGS data have provided huge support to predict the genes and drug targets in similar scenarios (39, 77, 78). Hence, in this study, we have attempted to integrate the whole genome transcriptomic data of independent LC- and EBV-infected PBMCs to detect the key DEGs. A total 357 cDEGs were detected between EBV and LC samples. As it is crucial to understand the interaction network of genes within the cell (79), these genes were subjected to PPI analysis using the STRING database (50). Out of 357 cDEGs, only 125 genes were involved in the interaction's formation (interacting score > 0.4). The rationale for considering cDEGs was to exclude sample-specific DEGs and focus on the shared transcriptomic profile, as this shared profile potentially reflects the regulation of common key biological pathways in the cell (80). Furthermore, we identified the 22 hub genes using the cystoscope plugin CytoHubba (52) and MCODE (54), followed by the enrichment analysis with ClueGo. Our enrichment results were consistent with previously reported studies (81). Since, during the disease condition, hub genes (because of being involved in the key functions) undergo substantial expression changes (82), we identified the individual gene expression under LC and EBV conditions. It was found that all 22 genes significantly changed (log2FC > 1 and *p*-value< 0.05) their expression in EBV and LC samples. TF enrichment analysis revealed that most of the hub genes were regulated by ZNF and MYB-family TFs. The involvement of these two TF classes has been previously reported in COVID (83) and EBV (84). As per our knowledge, there was not a single study available that utilized a similar multi-dimensional strategy to identify the pathogenic connection of LC and EBV infection. However, there is a vast range of COVID-19-related studies available such as Noor et al., which predicted the common pathogenic genes' profile between COVID-19 and HFRS (40). Luo et al. explored the common pathogenic mechanism between COVID-19 and primary Sjogren's syndrome (pSS) (77). In our study, the enrichment analysis revealed the role of hub genes in several immunological, neurological, and pulmonary system-related pathways. Most of the gene ontologies have already been reported in accordance with roughly COVID and EBV infections (28, 84–87). Because enrichment analysis comes from the biological intersection of LC and EBV DEGs, we expect their role in both LC and EBV infection symptoms simultaneously. These symptoms generally involved, the psychological problems/neurodegenerative disorders, or the terms associated with the brain function and the immunological disruption. Thus, there are existing lines of evidence suggesting that hub genes have a possible correlation with LC and EBV supported by previously published studies (15, 33, 88–90). Hub genes are functionally important and could act as very fine drug targets (91). Therefore, out of 22 hub genes, we choose one representative gene, OLR1 (oxidized low-density lipoprotein receptor 1), which was not studied previously with respect to LC-related manifestations. In COVID-19 patients, OLR1 gene positively expresses and causes cytokine storms and thrombosis (55). In severe COVID conditions, OLR1 is primarily involved in the activation of inflammatory immune responses (92). Considering the important role of hub genes in the cross-connection between LC and EBV reactivation, we used OLR1 protein as an important drug target and was docked against bioflavonoid inhibitors (70) The best five inhibitors, i.e., apigenin, amentoflavone, ilexgenin A, myricetin, and orientin, were screened out by virtual screening (93) based on their binding energies with OLR1. Several studies have reported the inhibitory and antiviral activities of these five compounds (72, 94–97).

Docking results provided great insight into the binding of shortlisted inhibitors with OLR1. Out of all five compounds, amentoflavone was considered as the best docked molecule with −8.3 kcal/mol energy, and the remaining compounds were in 6–7.7 kcal/mol range. Molecular docking gives insights into the ligand-receptor binding poses and pinpoints the bound amino acids with the ligands; however, it overlooks the strength and conformational modifications of the complex as well as the individual binding members that occur during the interaction's formation (98). The conformational stability of the complexes was further evaluated by MD simulations analyses, i.e., RMSD, RMSF, Rg, and hydrogen bonding. Comparative RMSD evaluation of ligand-bound and apo-OLR1 suggested the deviations' amplitude and the modest change in the average RMSD value of the C-backbone atoms, which clearly shows that the five OLR1 protein–ligand complex structures have a stable dynamic behavior. The comparative residual fluctuations of OLR1 were observed in both bound and apo form. N-terminus residues (start of protein) remain stable and did not exhibit any of the fluctuations until the 55th amino acid, followed by the fluctuations in between nearly 70 and 100 amino acids. Visual inspection of OLR1 protein suggested that this region mainly contains the loops that connect the alpha helices, and loops are considered as a variable structural component and they show a very dynamic behavior during the simulations (99, 100). Apart from the secondary structure, it was observed that the residues in the binding cavity also remained stable. Rg depicts the information about the compactness, shape, and folding of the four complex structures at various point scales throughout the 100 ns of MD simulations trajectory. The interaction between ligand and protein is influenced by non-covalent interactions including hydrogen bonding, hydrophobic forces, and ionic bonds. The quantity of hydrogen bonds and the duration for which they exist are key indicators of the stability and binding strength of the complexes (101). All five complexes exhibited an average four to six hydrogen bonds and remained stable throughout the course of 100-ns simulations. Binding free energy is another parameter to estimate the stability of a complex (102, 103). The binding free energy of each complex was calculated, and the overall order was amentoflavone > myricetin> orientin > ilexgenin A > apigenin with the total binding energy of −18.48, −14.13, −13.75, −11.07, and −9.55 kcal/mol, respectively. The lowest binding energy is proportional to the high stability of the complex (102); among all bound complexes, amentoflavone showed the lowest total binding energy. The highest positive correlation after the apo form was observed in the amentoflavone-bound complex, which indicates that, overall, the global changes between amino acids remained stable. The highest fluctuations (negative correlation) were observed in apigenin, myricetin, orientin, and ilexgenin A. Thus, all the MD analyses provided evidence that five inhibitors' compounds formed stable and dynamic conformations; however, amentoflavone was ranked as potentially the best inhibitor against OLR1 protein.

Although several previous independent studies have been carried out to explore the LC and EBV infection-related manifestations, there is room for studies that could predict the possible pathogenic biomarker genes and their inhibitors in the context of LC using integrative bioinformatics methods. Collectively, in this study, for the first time, we have attempted to explore and detect cDEGs, followed by hub genes and their associated TF identification using bioinformatics enrichment methods. Moreover, the hub gene protein (OLR1) interaction with bioflavonoids was performed using molecular docking and simulations. Among the five bioflavonoids, amentoflavone was predicted as the best inhibitor against OLR1.

However, it is important to acknowledge the potential limitations of this study. First, our findings are primarily based on publicly available transcriptomic datasets with a limited sample size. This may not be sufficient to identify all the key genes; thus, an increased sample size and sequencing depth could enhance the accuracy of predictions. Second, the statistical tools and models used to analyze the data may lead to overinterpretation, particularly when making inferences about causality or functional roles without adequate validation. Therefore, further *in vitro* or *in vivo* experiments would be valuable to functionally validate the hub genes and inhibitors predicted to target the hub protein in the clinical context of LC.

In summary, this study offers a valuable *in silico* framework for predicting the molecular links between EBV-mediated LC pathogenesis and may help identify potential marker genes with clinical significance, as well as inhibitors that could block the active sites of their corresponding proteins in LC. To sum up, our analysis predicted the possible genes and their pathways linking EBV reactivation and LC, elucidating some unknown clues in between. Nonetheless, as this is a thorough computational work, further case reports and follow-up experiments of LC patients can corroborate this association.

## 5 Conclusion

In summary, our results predicted 22 hub genes (CCL2, CCL20, CDCA2, CEP55, CHI3L1, CKAP2L, DEPDC1, DIAPH3, DLGAP5, E2F8, FGF1, NEK2, PBK, TOP2A, CCL3, CXCL8, DEPDC1, IL6, RETN, MMP2, LCN2, and OLR1) in the EBV infection and LC collective scenario with a significant change in expression. Through a series of comprehensive analyses, we predicted the biological roles of these genes in relation to EBV and LC. Our findings suggest that these genes are primarily involved in various immune-signaling pathways, including JAK-STAT signaling, interleukin signaling, protein kinase signaling, and toll-like receptor pathways, all of which are connected to LC. Furthermore, we propose an *in silico* framework to potentially uncover the clinical significance of hub genes associated with EBV infection in LC. Moreover, our results predicted the potential of hub genes as a drug target against bioflavonoids, which may serve as valuable therapeutic signatures in the clinical field upon further validation.

## Data Availability

The datasets presented in this study can be found in online repositories. The names of the repository/repositories and accession number(s) can be found in the article/**Supplementary Material**.
