# Supplementary material for: Exploring common pathogenic association between Epstein Barr virus infection and long-COVID by integrating RNA-Seq and molecular dynamics simulations
Source: Front Immunol. 2024 Sep 26;15:1435170. doi: 10.3389/fimmu.2024.1435170 (PMC11464307; doi:10.3389/fimmu.2024.1435170)
Supplement: Supplementary file 1 [file DataSheet1.docx]

Supplementary Material

**Exploring Common Pathogenic Association between Epstein Barr Virus Infection and Long-COVID by Integrating RNA-Seq and Molecular Dynamics Simulations**

Ayesha Kanwal and Zhiyong Zhang*

*** Correspondence:** Zhiyong Zhang: [zzyzhang@ustc.edu.cn](mailto:zzyzhang@ustc.edu.cn\)

# Supplementary Figures and Tables

## Supplementary Figures


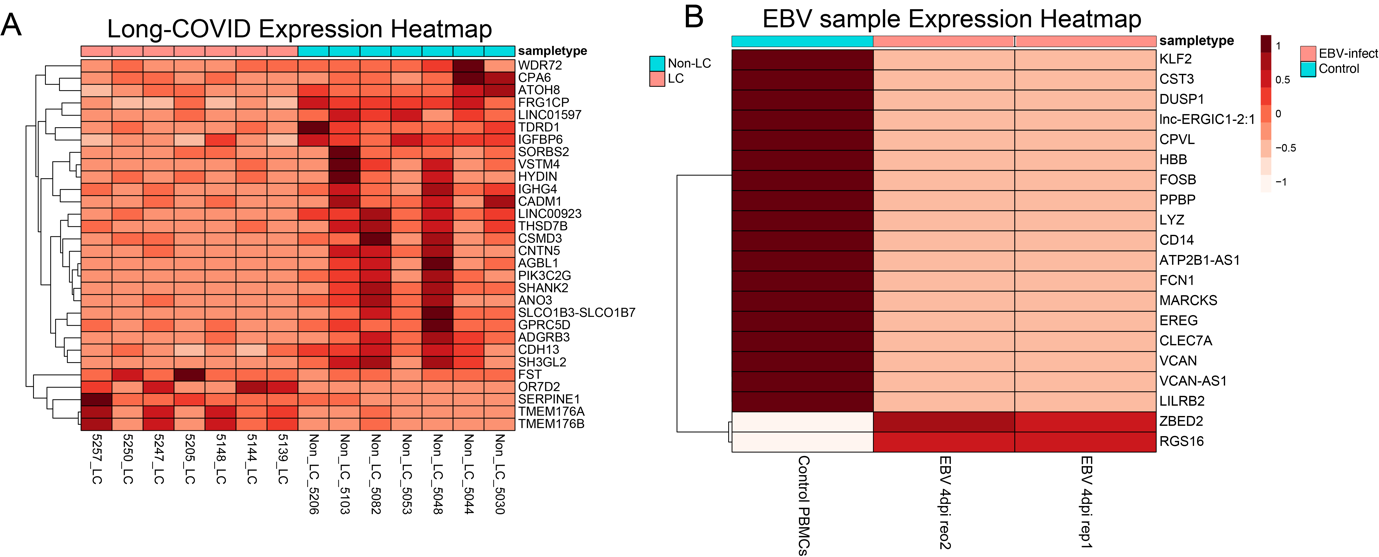


**Supplementary Figure S1.** **Gene expression heatmap of Long-COVID and EBV samples DEGs.**

**A)** DEGs expression of Long-COVID samples. **B)** DEGs expression of EBV samples. DEGs are visually represented in the matrices color represented the level of expression that range from (Log2FoldChange > 1 and p-value <0.05). The samples names are represented on X-axis and genes names are plotted in the Y-axis. The hierarchical clustering of the genes is shown in both heatmaps.


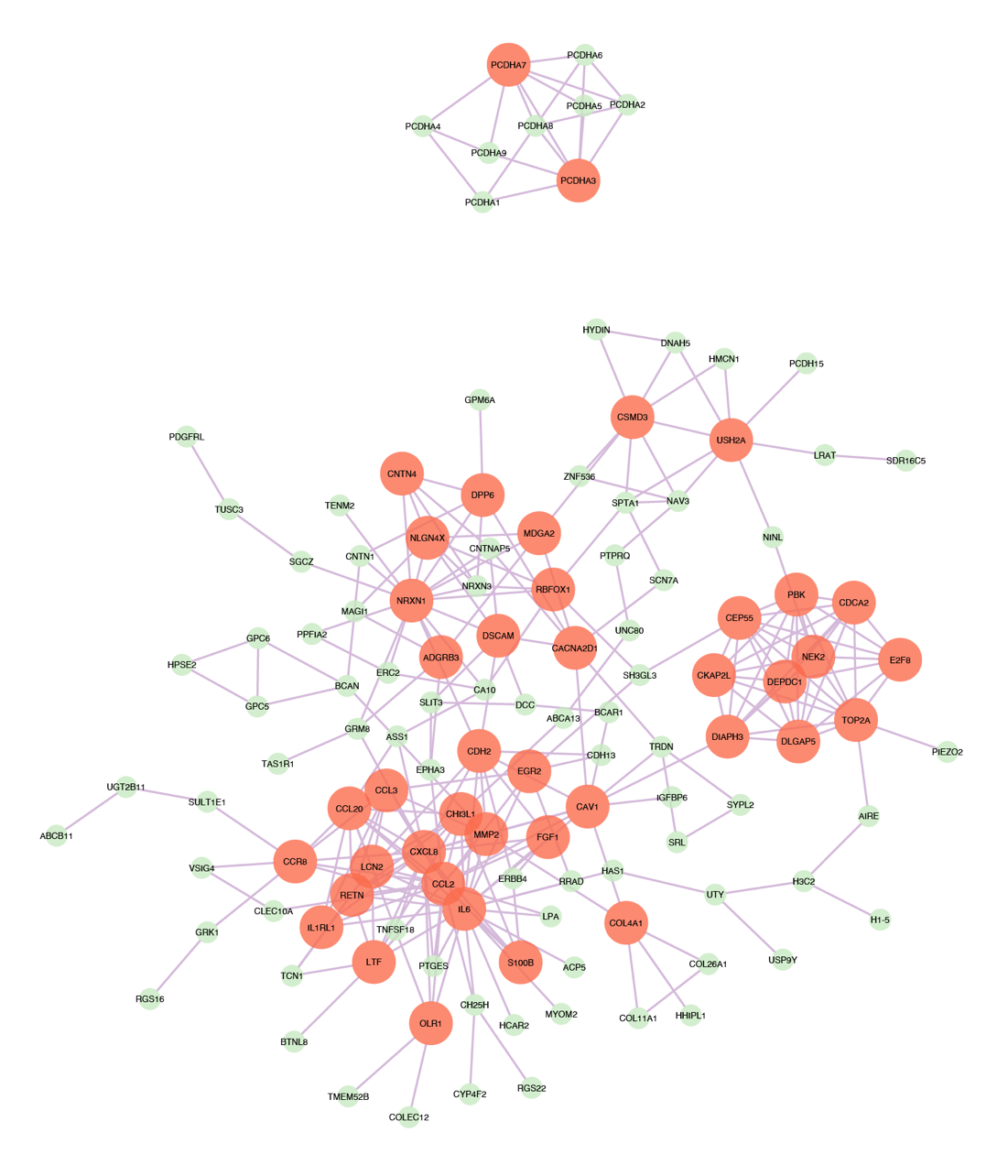


**Supplementary Figure S2. Network analysis of Common Genes between Long-COVID and EBV samples**

Common DEGs expression of Long-COVID samples. **B)** DEGs expression of EBV samples. DEGs are visually represented in the matrices color represented the level of expression that range from (Log2FoldChange > 1 and p-value <0.05). The samples names are represented on X-axis and genes names are plotted in the Y-axis. The hierarchical clustering of the genes is shown in both heatmaps.


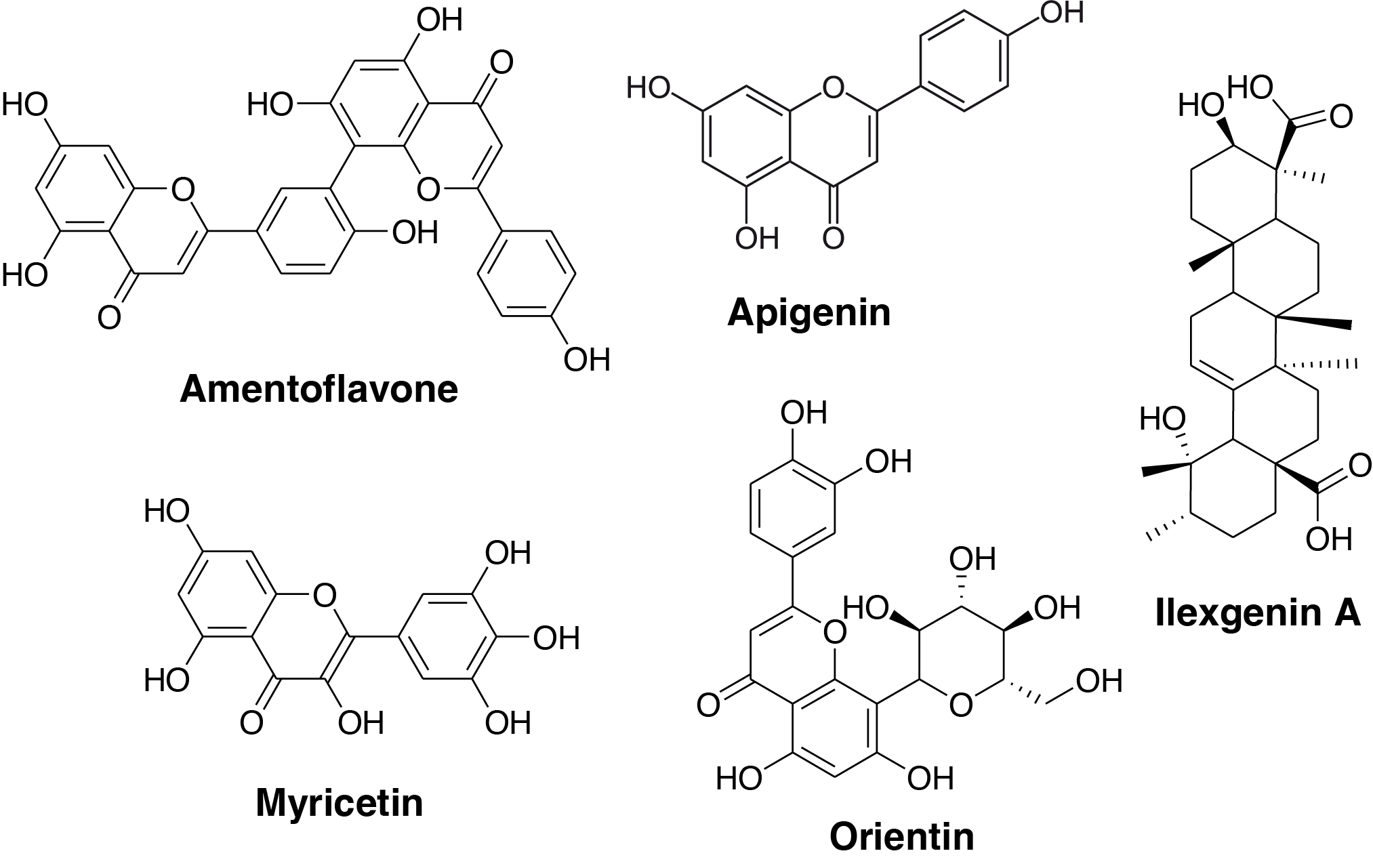


**Supplementary Figure S3. Small molecules against hub-gene OLR1 protein**

Total five best small molecules belong to bioflavonoids class used to dock against OLR1 protein.


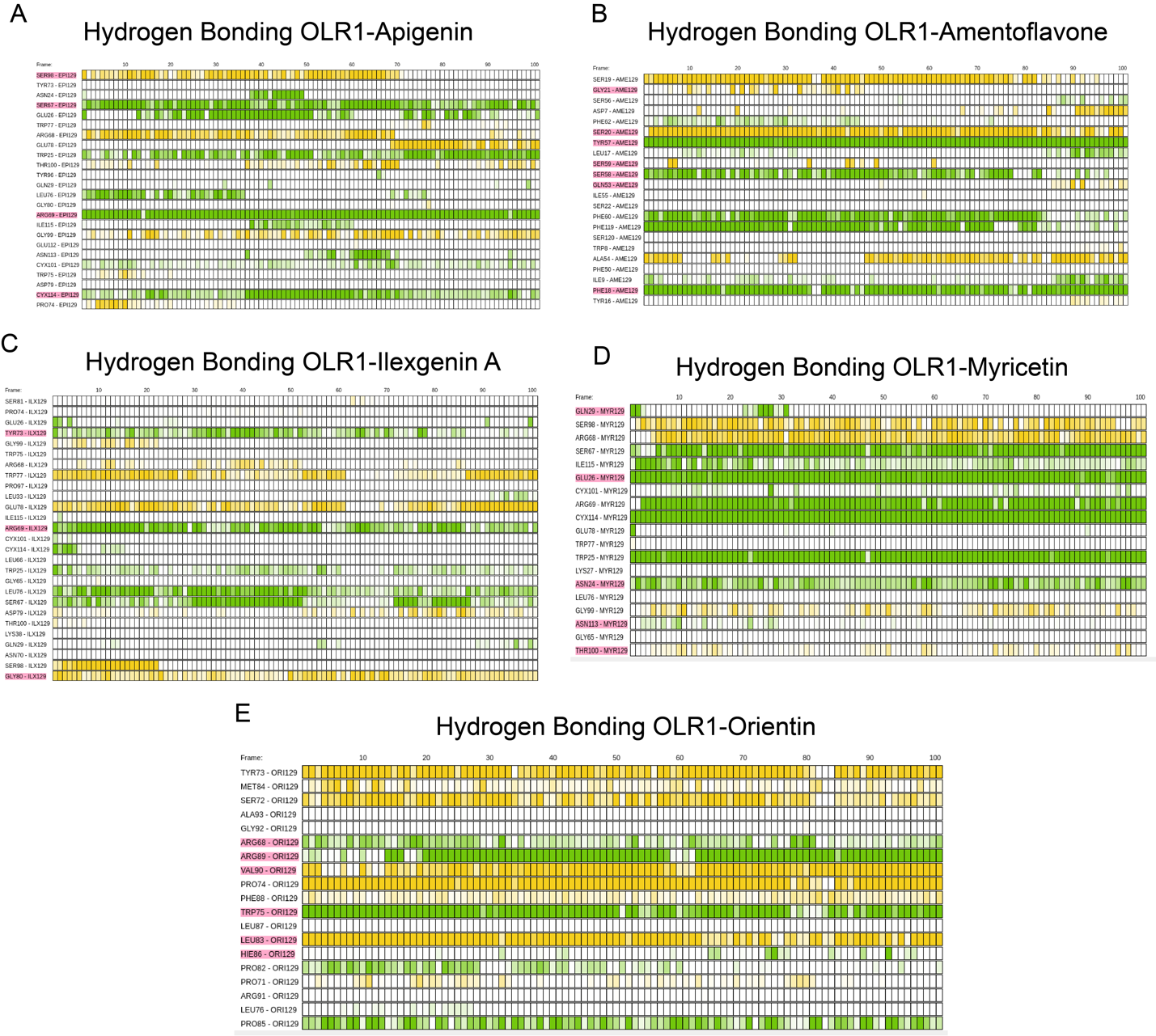


**Supplementary Figure S4. OLR1-protein residue involved in hydrogen bonds formation.**

The residues involved in hydrogen bond formation are presented for each ligand and OLR1 protein complex. **A)** OLR1-Apigenine, **B)** OLR1-Amentoflavone, **C)** OLR1-Ilexgenin A, **D)** OLR1-Myricetin and, **E)** OLR1-Orientin.Yellow color bar represents the backbone/side chain hydrogen bonds. Green color indicates the side chain/side chain hydrogen interaction. The strength of bonding is shown by the color gradient i:e weak bonds with faded color and strong bonds with darker color. The purple color labeling on the X-axis representing the “True” hydrogen bonds.


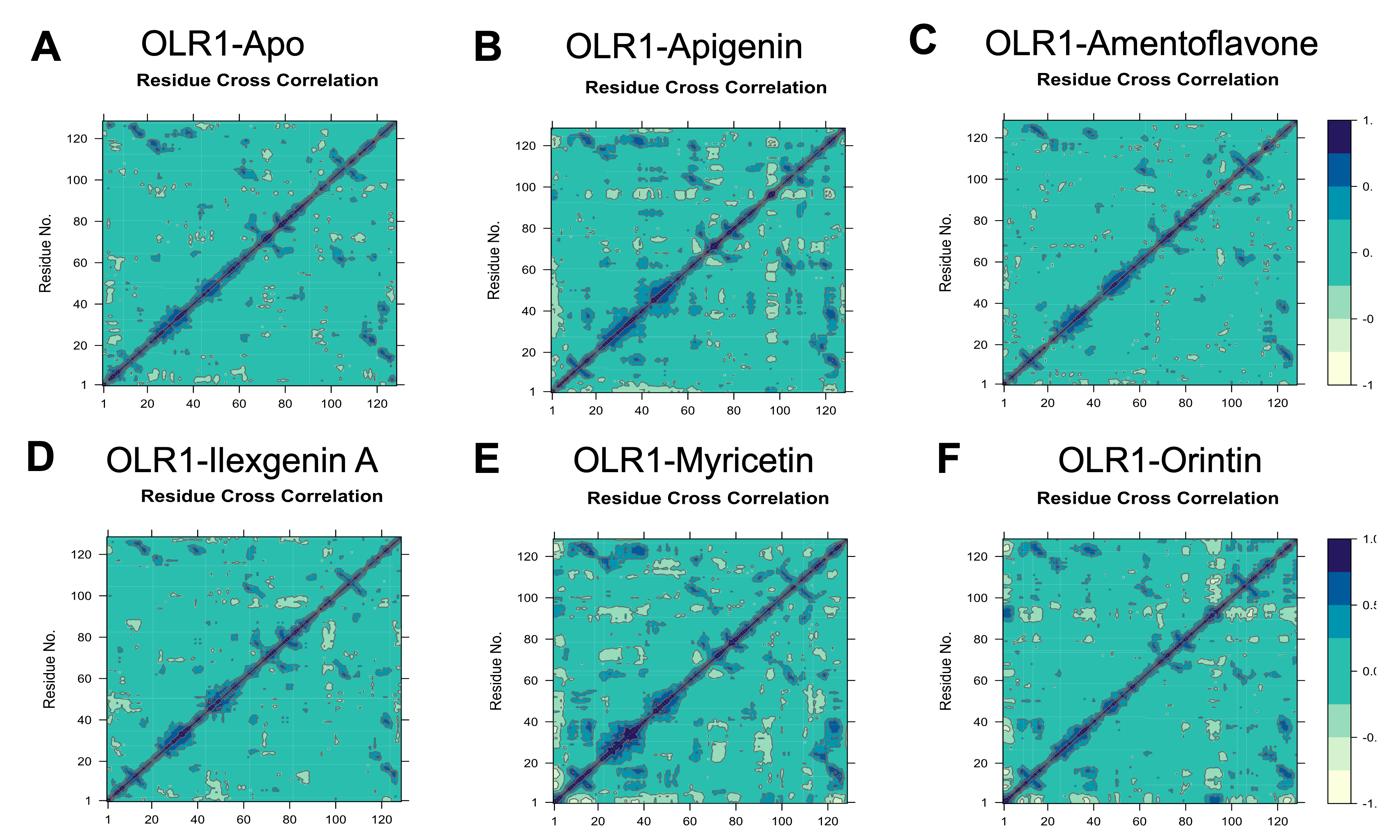


Supplementary Figure S5. Residual cross correlation of OLR1-Apo and ligand bounded complex

Cross correlation between residues of Apo and ligand bounded complex has been plotted in the correlation matrix. **A)** OLR1-Apo **(B)** Apigenin, **C)** Amentoflavone, **D)** Ilexgenin A, **E)** Myricetin and **F)** Orientin has been presented. Yellow color is corresponding to the negative correlation and blue color is indicating extreme positive correlation.


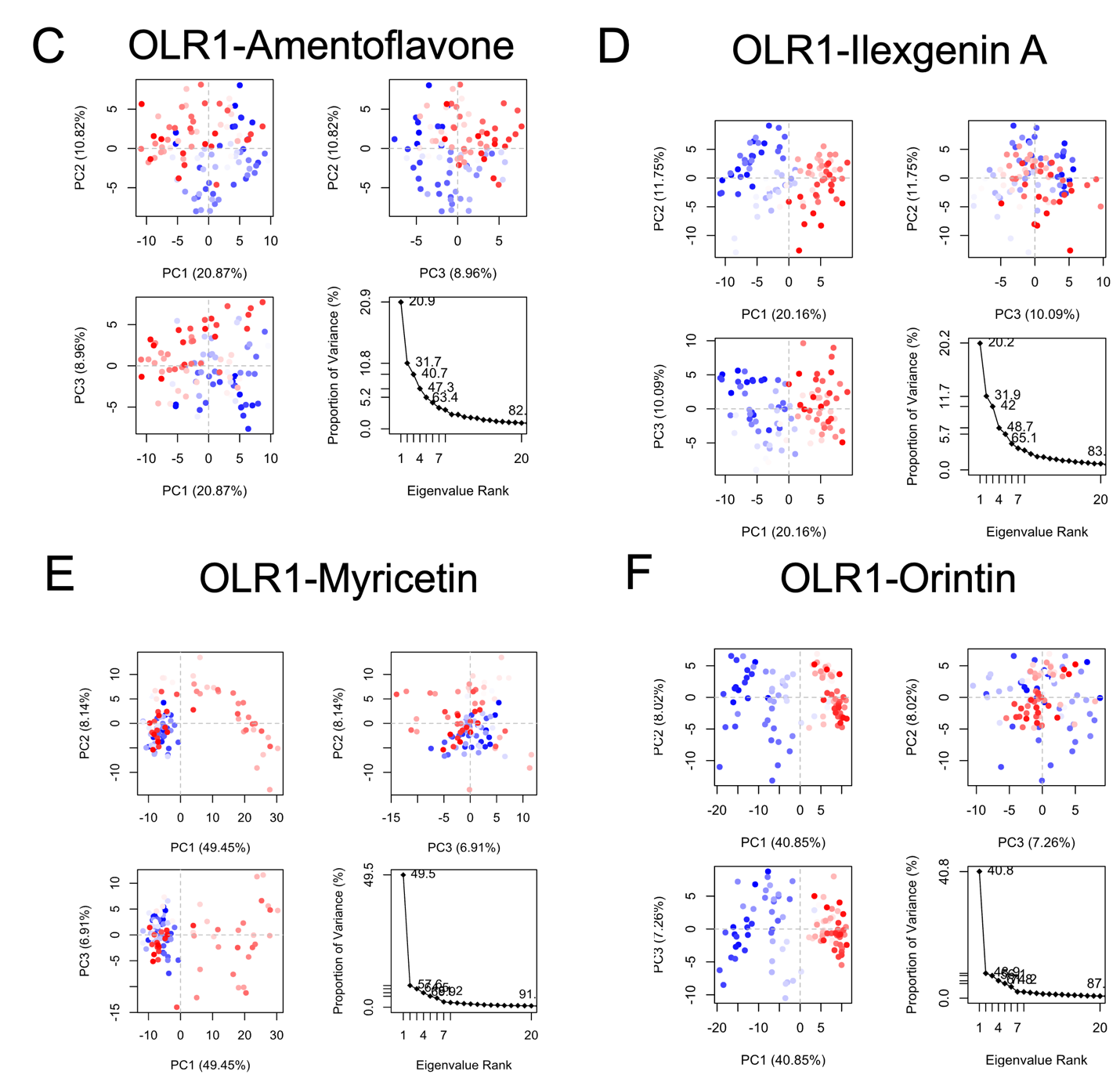


**Supplementary Figure S6. Principal component analysis (PCA) or remaining ligands bounded OLR1.**

**C)** Amentoflavone, **D)** Ilexgenin A, **E)** Myricetin and **F)** Orientin has been presented. PCA values are calculated, and the calculated variance was calculated and presented in each panel. PC1, PC2, PC3 and Eigenvector ranks are shown in each panel respectively.

## Supplementary Tables

Supplementary Table S1. Input RNA-Seq data details.

| SRA ID | Normal data | SRA ID | Diseased data |
| --- | --- | --- | --- |
| SRR23348798 | 5206_Non-LC_WT | SRR23348793 | 5257_LC_WT |
| SRR23348809 | 5103_Non-LC_WT | SRR23348794 | 5250_LC_WT |
| SRR23348813 | 5082_Non-LC_WT | SRR23348795 | 5247_LC_WT |
| SRR23348819 | 5053_Non-LC_WT | SRR23348799 | 5205_LC_WT |
| SRR23348821 | 5048_Non-LC_WT | SRR23348801 | 5148_LC_WT |
| SRR23348822 | 5044_Non-LC_WT | SRR23348802 | 5144_LC_WT |
| SRR23348825 | 5030_Non-LC_WT | SRR23348804 | 5139_LC_WT |
| SRR25040732 | Control PBMCS | SRR25040729 | 4^th^ day Rep1  EBV |
| ----- | ----- | SRR25040728 | 4^th^ day Rep2  EBV |

Supplementary Table S2. Mapping stats of normal and Diseased samples.

| Sample ID | Total reads | Mapped reads | Percentage | Sample type |
| --- | --- | --- | --- | --- |
| SRR23348798 | 47817356 | 44217979 | 92.47% | Non-LC |
| SRR23348809 | 41618613 | 38508632 | 92.53% | Non-LC |
| SRR23348813 | 47394028 | 43921092 | 92.67% | Non-LC |
| SRR23348819 | 51573096 | 47638870 | 92.37% | Non-LC |
| SRR23348821 | 42644040 | 39642175 | 92.96% | Non-LC |
| SRR23348822 | 47960877 | 44100818 | 91.95% | Non-LC |
| SRR23348825 | 46567404 | 43359594 | 93.11% | Non-LC |
| SRR23348793 | 43296606 | 39859652 | 92.06% | LC |
| SRR23348794 | 42942477 | 39856337 | 92.81% | LC |
| SRR23348795 | 47226107 | 43753735 | 92.65% | LC |
| SRR23348799 | 44611652 | 41311917 | 92.60% | LC |
| SRR23348801 | 46891883 | 43246604 | 92.23% | LC |
| SRR23348802 | 42716361 | 39603804 | 92.71% | LC |
| SRR23348804 | 47317776 | 44259032 | 93.54% | LC |
| SRR25040732 | 23091469 | 21469651 | 92.98% | Control PBMCs |
| SRR25040729 | 20985527 | 19468602 | 92.77% | 4^th^ day Rep 1  EBV-infection |
| SRR25040728 | 23169346 | 21145273 | 91.26% | 4^th^ day Rep2  EBV-infection^[[1]](#footnote-1)^ |

Supplementary Table S3. Binding free energy (Kcal/mol) components of ORL1 protein with all compounds.

| Energy | Apigenin | Amentoflavone | Ilexgenin A | Myricetin | Orientin |
| --- | --- | --- | --- | --- | --- |
| ΔEvdw | -20.51 | -28.36 | -21.46 | -27.05 | -28.92 |
| ΔEelec | -6.87 | -15.60 | -13.85 | -5.54 | -22.24 |
| ΔGGAS | -27.38 | -43.96 | -35.31 | -32.59 | -51.16 |
| ΔGSOLV | 17.83 | 25.49 | 24.24 | 18.45 | 37.41 |
| ΔTOTAL | -9.55 | -18.48 | -11.07 | -14.13 | -13.75 |

Supplementary Table S4. Predicted Hub genes between Long-COVID and EBV-infected samples.

Attached Excel file.

1. Long-COVID (LC) [↑](#footnote-ref-1)
